# Insights into the Bead Fusion Mechanism of Expanded Polybutylene Terephthalate (E-PBT)

**DOI:** 10.3390/polym13040582

**Published:** 2021-02-15

**Authors:** Justus Kuhnigk, Daniel Raps, Tobias Standau, Marius Luik, Volker Altstädt, Holger Ruckdäschel

**Affiliations:** 1 Department of Polymer Engineering, University of Bayreuth, Universitätsstraße 30, 95447 Bayreuth, Germany; justus.kuhnigk@uni-bayreuth.de (J.K.); daniel.raps@gmx.net (D.R.); tobias.standau@uni-bayreuth.de (T.S.); marius.luik@uni-bayreuth.de (M.L.); volker.altstaedt@uni-bayreuth.de (V.A.); 2 Bavarian Polymer Institute and Bayreuth Institute of Macromolecular Research, University of Bayreuth, Universitätsstraße 30, 95447 Bayreuth, Germany

**Keywords:** bead foam, fusion mechanism, crystallization, expanded polybutylene terephthalate, E-PBT, crystallization kinetics, Avrami, steam chest molding, chain extender, fusion

## Abstract

Expandable polystyrene (EPS) and expanded polypropylene (EPP) dominate the bead foam market. As the low thermal performance of EPS and EPP limits application at elevated temperatures novel solutions such as expanded polybutylene terephthalate (E-PBT) are gaining importance. To produce parts, individual beads are typically molded by hot steam. While molding of EPP is well-understood and related to two distinct melting temperatures, the mechanisms of E-PBT are different. E-PBT shows only one melting peak and can surprisingly only be molded when adding chain extender (CE). This publication therefore aims to understand the impact of thermal properties of E-PBT on its molding behavior. Detailed differential scanning calorimetry was performed on neat and chain extended E-PBT. The crystallinity of the outer layer and center of the bead was similar. Thus, a former hypothesis that a completely amorphous bead layer enables molding, was discarded. However, the incorporation of CE remarkably reduces the crystallization and re-crystallization rate. As a consequence, the time available for interdiffusion of chains across neighboring beads increases and facilitates crystallization across the bead interface. For E-PBT bead foams, it is concluded that sufficient time for polymer interdiffusion during molding is crucial and requires adjusted crystallization kinetics.

## 1. Introduction

In contrast to foam extrusion and foam injection molding, the bead foaming techno-logy is a manufacturing process which involves molding and sintering of tiny foamed beads into plastic foam components. In this aspect, the bead foaming procedure is consi-dered to be a highly promising technology, which allows both the high foam expansion of extrusion foaming and the geometrical complexity of the resulting part in the foam injection molding process [1,2,3,4]. The most important representatives of bead foams in terms of volume are expandable polystyrene (EPS) [5,6], which is widely used in packaging, heat and sound insulation, expanded polyethylene (EPE) [7] and expanded polypropylene (EPP) [8,9], which is used in applications such as packaging, cushioning, exterior and interior automotive parts and many other commodity applications. There are also developments in foams made of thermoplastic polyurethane (ETPU) [10,11,12] and polylactide (EPLA) [13,14,15,16]. However, the previous mentioned matrix polymers have the disadvantage of a low long term heat resistance temperature (80 °C for EPS, 110 °C for EPP), thus excluding certain processes (e.g., cathode dip coating, sandwich consolidation under increased pressure and/or temperature as in the RTM process) or applications in the engine compartment, in so-called under-the-hood applications, such as for ducts and covers. Presently, the first commercial approaches for the realization of bead foams with higher continuous service temperatures are already available, including PMI (Evonik AG) [17], PET (Armacell S.A.) [18] and PA (BASF SE, Asahi Kasei K.K.) [19,20] PESU (BASF SE) [21].

In addition to these already established bead foams, research is also done on the development of temperature-resistant bead foams made of polybutylene terephthalate (PBT) in a continuous process. In our previous work the production of PBT bead foams was described for the first time. The study by Köppl et al. [22] focused on important parameters in terms of expansion and morphology of the beads that were produced in a continuous process by coupling foam extrusion and underwater pelletizing. The importance of the viscosity on the foam morphology was clearly shown. Thus, with a higher viscosity, a finer cell morphology and a rounder shape of the bead foams could be achieved. However, it was not possible to weld them into a molded part. In a subsequent work by Standau et al. [23] it was shown that by chemical modification with a multifunctional epoxy containing chain extender (CE) not only improved foamability but also for the first time weldability of the PBT beads into a molded part could be achieved. Usually the production of foamed beads into a finished part is done in a steam chest molding process, which is sufficiently explained in the literature [1]. The energy introduced by the steam leads to a superficial fusion of the beads, while the foam structure inside remains intact. During molding, chain inter-diffusion across the bead interfaces occurs in a thin layer on the surface of two touching foamed beads resulting in a strong physical bond [24,25,26,27]. To reduce the viscosity and increase the polymer chain mobility the molding temperature is set above the glass transition temperature *T_g_* (amorphous polymers) or above the melting temperature *T_m_* (semi-crystalline polymers), respectively [28]. The phenomenon of inter-diffusion across a surface is well described by several authors in the context of welding or healing of interfaces and cracks [1,24,26,27,28]. The subsequent cooling cycle in the steam chest molding process freezes the physical entanglement and causes dimensional stability of the molded part.

An explanation why unmodified PBT bead foams cannot be welded into a molded part, while weldability can be achieved by chain extension (CE), has not yet been provided. The sintering process E-PBT bead foams differs significantly from the fusion mechanism of the established bead foams. In the case of amorphous bead foams such as EPS the superheated steam causes the individual beads to heat up above the glass transition temperature *T_g_*. As a result, chain mobility increases and interdiffusion and entanglement of the polymer chains across the bead interfaces becomes possible [29,30]. Semi-crystalline polymers such as EPP have two melting peaks due to the autoclave foaming process. An isothermal saturation step in the autoclave at elevated temperature leads to the formation of a high-temperature melting peak with a more perfect crystal structure. During cooling the low melting peak is formed. The steam temperature is set between the two melting peaks, so the foam structure is stabilized by the non-molten crystallites with the higher *T_m_*. The molten crystals of the low melting peak contribute to interdiffusion and sintering of the beads as the temperature is far above *T_g_* [1,9,14,31,32].

Neither of the two described bead fusion mechanisms applies to E-PBT. PBT is semi-crystalline and for the bead foams only one melting peak was observed. E-PBT modified by multifunctional epoxy containing chain extender (CE) fuses well below the melting temperature of PBT but above the glass transition temperature *T_g_*. This contradicts the current knowledge about molding of semicrystalline bead foams (e.g., EPP). In a hypothesis made by Standau et al. [23] it was assumed that the bead skin gets quenched during underwater pelletizing when the expanding hot PBT melt (220 °C) comes into contact with the much colder water flow (80 °C). This would result in an amorphous skin which could be beneficial for the sintering process. However, regarding crystallinity, no significant differences between surface and core of PBT bead foams could be identified yet.

Aim of the present work was to gain insights into the bead fusion mechanism in E-PBT through investigation of the melting and crystallization behavior of E-PBT by diffe-rential scanning calorimetry. For this purpose non-isothermal and two different isothermal crystallization routes at various crystallization temperatures for neat and 1 wt. % CE modified E-PBT were carried out. In particular, the isothermal cold-crystallization behavior were investigated to understand the process during molding. Activation energies and kinetic crystallization parameters were obtained through Avrami evaluations and compared with each other to work out the influence of chain extender (CE) modification on the crystallization behavior and thus its role for the molding process.

## 2. Materials and Methods

In this study, PBT Pocan B 1300 from Lanxess AG (Cologne, Germany) and the multi-functional epoxy-based chain extender (CE) Joncryl^®^ 4468 from BASF SE (Ludwigshafen, Germany) was used. The polymer was dried for 4 h at 80 °C prior to use. Bead foams were produced as described in one of our former studies [23] with a Dr. Collin tandem foam extrusion line (Ebersberg, Germany) coupled with an under-water granulator LPU from company Gala Kunststoff- und Kautschukmaschinen GmbH (Xanten, Germany). Trials were carried out with the same machine settings as before and with 2 wt. % CO_2_ as blowing agent and 1 wt. % CE for melt modification.

Differential scanning calorimeter (DSC) measurements were conducted to generate local information, for example only of the welding relevant outer layer. Moldable and not moldable beads were skinned for this trials.

The thermal behavior of the pure E-PBT and chain extender modified E-PBT beads were determined by the use of a differential scanning calorimeter (DSC 1) from Mettler Toledo (Columbus, OH, USA). In the case of non-isothermal crystallization studies, samples (between 5 and 8 mg) were heated in nitrogen atmosphere in a temperature range from 25 to 270 °C at a heating rate of 10 K/min and cooling rates of 2.5, 5, 10 and 20 K/min. To guarantee the reproducibility of the results, all measurements were carried out three times. The degree of crystallinity was determined with the theoretical value for the heat of fusion of 100% crystal PBT ($\Delta H_{m}^{0}$ = 140 J/g) from literature [33]. The evaluation was carried out with the STARe-software (Mettler-Toledo AG, Schwerzenbach, Switzerland) according to Khanna et al. [34] with a straight base line. The analysis of the non-isothermal crystallization kinetics for the DSC results was performed according to Jeziorny-modified Avrami theory [35].

The DSC experiments of isothermal crystallization of pure E-PBT and chain extender modified E-PBT beads were also determined by the use of a differential scanning calorimeter (DSC 1) from Mettler Toledo (Columbus, OH, USA). Depending on the different routes to crystallization temperatures the samples were cooled from the melt or heated from the glass and designated as isothermal hot- and cold-crystallization. In the case of isothermal hot-crystallization, the samples (between 5 and 8 mg) were heated to melt from 25 to 250 °C at a rate of 50 K/min, and then cooled to the desired crystallization temperature (*T_c_*) at a rate of 50 K/min and maintained at *T_c_* for isothermal crystallization tests. For the isothermal cold-crystallization studies the samples were heated from 25 °C to the desired crystallization temperature (*T_c_*) at a rate of 50 K/min and maintained at *T_c_* for isothermal crystallization tests. For both isothermal hot- and cold-crystallization the *T_c_* was set for *T*_1_ (190 °C), *T*_2_ (195 °C), *T*_3_ (200 °C) and *T*_4_ (205 °C). Each result is an average of three.

The analysis of the isothermal crystallization kinetics for the DSC results was performed according to Avrami theory [36,37]. Since Avrami plots strongly depend on the shape and position of the borders of the crystallization process and in order to guarantee reproducibility the start and the end of crystallization window were determined according to the recommendations of Lorenzo et al. [38].

## 3. Results and Discussion

### 3.1. Crystalline Propertis of PBT Bead Foams

**Figure 1:**
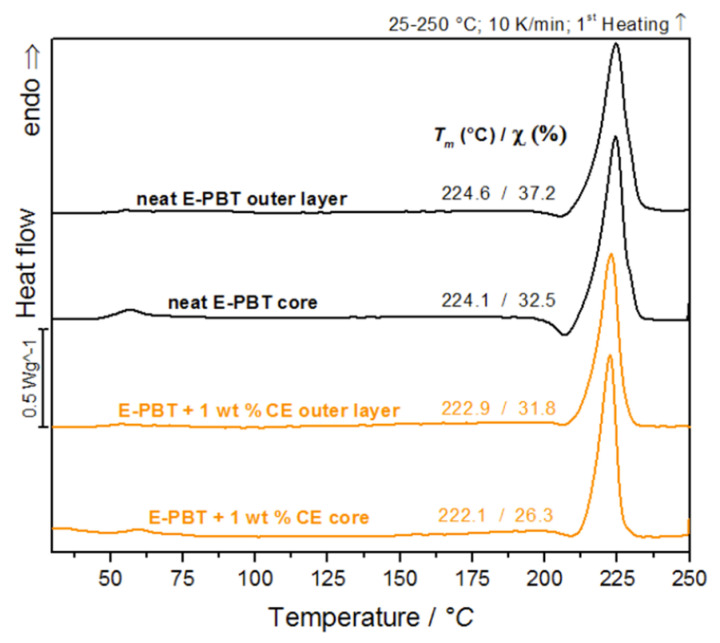
DSC-analysis of bead outer layers and cores of neat E-PBT and 1 wt. % CE modified E-PBT (first heating curve is shown).

The classical bead fusion explanations, as for EPS or EPP, seem to be invalid in case of E-PBT as explained before. The study of the outer layer of the manufactured PBT bead foams forms the basis for understanding the processes that occur during the molding of the beads. The following crystallinity studies are based on the hypothesis that quenching during the underwater pelletizing process creates a more amorphous outer layer. Therefore it is essential to investigate how the crystallinity on the bead surface differs from that in the core. The first heating curves were analyzed to determine the influence of the processing. Figure 1 compares the DSC curves of the respective thin outer layer and core of the unmodified and 1 wt. % CE modified PBT beads. The DSC investigations on outer layer compartment and the core seem to be evidence that there are differences in the crystallization behavior from the inside to the outside. Table 1 compares the determined melting temperature *T_m_*, melting enthalpy Δ*H_m_* and relative crystallinity χ of the DSC thermograms shown in Figure 1.

After leaving the nozzle, the internal CO_2_ gas pressure of the foam cells causes an equibiaxial expansion of the surrounding melt. As a result, the polymer chains are oriented and stretched in the direction of expansion, which promotes strain-induced crystallization [39,40]. It can be assumed that in a bead foam, the extent of strain-induced crystallization is most pronounced on the bead skin and that a crystallization gradient is formed within the bead, which increases from the inside (core) to the outside (skin), when thermal effects are ignored. The fact that the content of crystallization in the CE modified PBT beads is less pronounced in both the core and outer layer regions (compared to the unmodified beads) can be explained as follows. In previous extensional rheological investigations it could be shown that the unmodified PBT material does not show strain hardening behavior compared to the CE modified bead [23]. Since the resistance to elongation increases due to the strain hardening, the extent of strain-induced crystallization (which reflects the degree of orientation of the polymer chains) should also be suppressed for the CE modified material. The occurrence of strain hardening suggests that CE modification induces branching in the PBT chain architecture which is also known for other polymers [41,42]. Due to the bulky groups in the CE structure [41] and increased branching degree, the steric demand in the PBT polymer increases [43,44]. This reduces the chain mobility so that the formation of crystals in the branched PBT system is reduced compared to the linear one [45]. It also can be noted that the CE modified PBT beads (both core and outer layer) have less pronounced cold-crystallization peaks in the 1st heating curve (see Table 1) compared to the unmodified beads. This observation is an indication for the reduced chain mobility caused by CE-induced branching.

**Figure 2:**
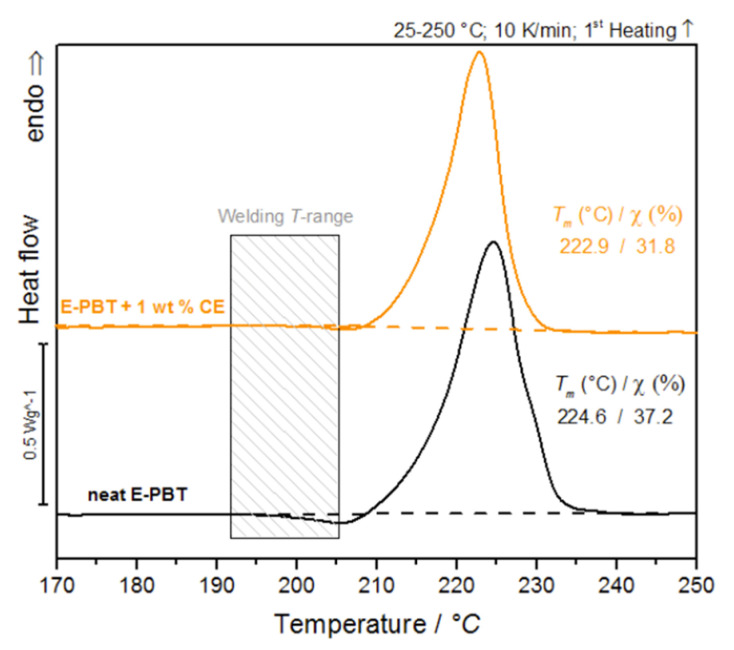
DSC-analysis of bead outer layers of neat E-PBT and 1 wt. % CE modified E-PBT (first heating curve).

Since the formation of inter-bead bonding involves the diffusion of polymer chains across the interfaces between the beads, the crystalline properties of the outer layers (unmodified and CE modified) are considered for further clarification of the fusion mechanism in PBT bead foams (Figure 2).

It is obvious that the cold-crystallization peaks of the PBT beads (unmodified and CE modified) occur in the welding temperature range. The previous considerations regarding the influence of branching on the crystallization behavior give rise to a new hypothesis: PBT without chain extension or a short retention time leads to a fast crystallization during steaming and the beads do not fuse while chain extension (branching) reduces the total crystallization and crystallization rate during molding so that the PBT can beads fuse together.

### 3.2. Non-Isothermal Crystallization Kinetics

**Figure 3:**
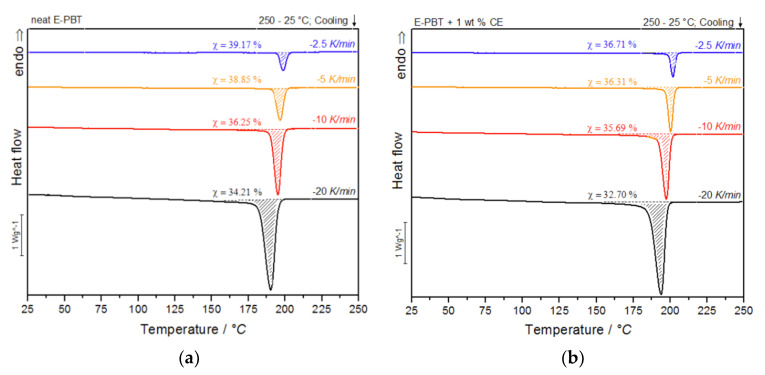
DSC thermograms of (**a**) neat and (**b**) CE modified E-PBT at cooling rates of −2.5, −5, −10 and −20 K/min.

During the under-water granulation, the expanding PBT melt (approximately 220 °C) comes in contact with the much colder water (80 °C). These high cooling rates cannot be simulated with standard DSC measurements and therefore can only be assumed, based on lower possible cooling rates. Nevertheless, insights into the crystallization behavior of PBT during the bead foam process can be obtained via non-isothermal crystallization measurements and the dependence on different cooling rates can be worked out. In Figure 3a,b the DSC cooling thermograms of the neat and CE modified PBT beads are shown.

**Figure 4:**
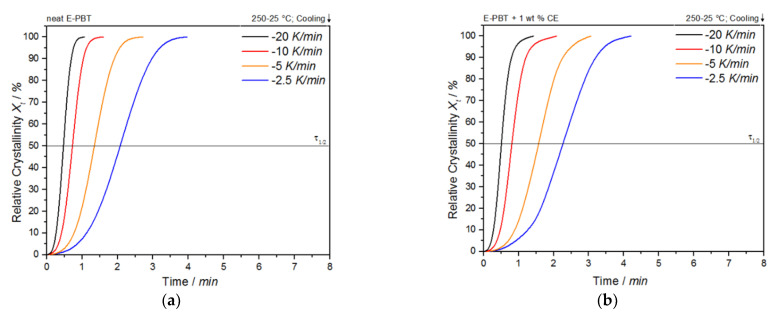
Conversion of the crystallization process at different cooling rates for (**a**) neat and (**b**) CE modified E-PBT.

As shown in Figure 3a,b, the temperature range (Table 2) for crystallization shifts for both neat and CE modified E-PBT towards lower temperatures when the cooling rate ϕ increases. Furthermore, it can be seen (Figure 3, Table 2) that for all considered cooling rates ϕ, the crystallization of the CE modified E-PBT is less pronounced, the crystallization process starts at higher temperatures and the required time for complete crystallization is longer than for the unmodified PBT beads. The earlier start of crystallization for the CE modified E-PBT could be explained by a nucleation effect of the branches induced by the CE reaction [45,46]. With the non-isothermal crystallization exotherms the conversion of the relative crystallinity (*X_t_*) at different crystallization times can be determined. The time variable can be calculated with the temperature and the cooling rate according to Equation (1), where *T*_0.1%_ is the temperature when 0.1% of the crystallization occurs (*t* = 0). The resulting plots can be seen in Figure 4a,b.

(1) $$t=\frac{T_{0.1\%}-T}{\phi }$$

The curves all show typical sigmoidal dependence with time and are linear from 10 to 90%. A steeper slope of the linear portion represents a higher rate of crystallization. The required time for complete crystallization for both neat and CE modified E-PBT becomes longer with decreasing cooling rate ϕ. The dependence of the total crystallization rate on the crystallization temperature is particularly evident in the half-lives τ_1/2_, which is defined as the time at which the relative degree of crystallinity reaches 50%. The half-time of crystallization τ_1/2_ as function of ϕ obtained from Figure 4 is listed in Table 2 for neat and CE modified E-PBT.

The peak width as well as the half-time of crystallization are inversely dependent on each other. Clearly, it can be seen that the half-life time τ_1/2_ decreases with increasing coo-ling rate ϕ for both neat and CE modified E-PBT. Due to the existing branches in the CE modified E-PBT the crystallization starts at higher temperatures, but also the overall crystallization process is hindered, which can be seen in greater crystallization half times τ_1/2_ for all considered cooling rates ϕ. These observations are not new and already stated for other polymers in the literature [45,46], but have never been shown for bead foams. Further kinetic investigations of the non-isothermal crystallization were analyzed on the basis of the following Avrami equation [36,37]:

*X_t_* = 1 − exp (−*k^1^*^/*n*^*t*)*^n^* (2)

where *k* is the crystallization rate constant and *n* is the Avrami exponent which describes the dimensionality of nucleation and growth. After conversion, Equation (2) takes the well-known form:

ln[−ln(1 − *X_t_*)] = *n* ln*t +* ln*k* (3)

Usually the Avrami approach is used to describe the isothermal crystallization behavior. However, this model neglects the influence of the cooling rate and the thermal gradient on the polymer sample. Non-isothermal crystallization kinetics can be described by the modified Avrami equation by Jeziorny [35] which takes the heating/cooling rates ϕ into account. For the kinetic crystallization rate *k_c_* follows:

(4) $$\ln k_{c}\;=\;\ln\frac{k}{\phi }$$

**Figure 5:**
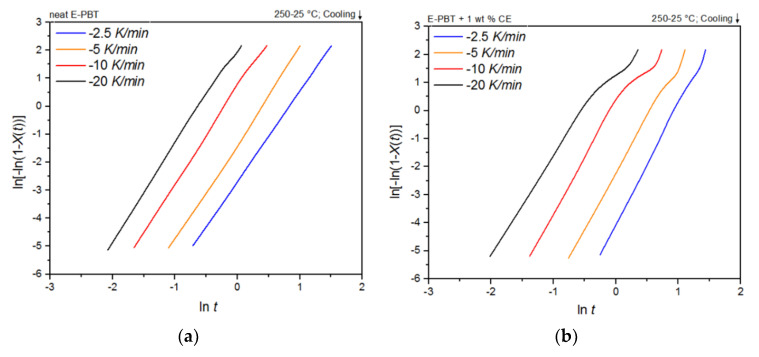
Avrami analysis for (**a**) neat and (**b**) 1 wt. % CE modified E-PBT at different cooling rates ϕ.

Plots of ln[–ln(1–*X_t_*)] versus ln*t* at given temperatures *T_c_* yields a straight line for neat and CE modified E-PBT which is shown in Figure 5a,b. According to Equation (3), values of *n* and *k* are obtained from the slope and the point of intersection with the *y*-axis and values for the corrected crystallization rate constant *k_c_* related to the corresponding cooling rate ϕ are given by Equation (4) and listed in Table 3. For the neat E-PBT each plot shows characteristic linear behavior for all cooling rates ϕ reflecting the primary crystallization stage of the samples. Only at a high cooling rate of 20 K/min at a high degree of crystallization a very slight deviation from linearity can be observed due to the secondary crystallization. This non-linear behavior due to the secondary crystallization is much more pronounced for the CE modified E-PBT and increases with higher cooling rates ϕ and is already known from the literature [47,48]. It can be assumed that with the much higher cooling rates during the bead foam process, secondary crystallization is much more pronounced, especially for the CE modified E-PBT. The kinetic constants by Jeziorny modification calculated from the linear portion are listed in Table 3. The variation in the Avrami constants *n* between neat and the CE modified E-PBT shows that the introduction of branches has effects on nucleation and crystal growth of PBT crystallites. For the neat E-PBT, the *n* values vary in the range between 3.23 and 3.46 (which is in accordance to literature [49,50,51], for the CE modified E-PBT in the range of 3.95 and 4.28. The higher average *n* value of 4 in the case of the CE modified E-PBT shows that the branches induced by the CE reaction have an additional heterogeneous nucleation effect (constant nucleation rate) and promote three-dimensional spherulitic crystal growth. [47,49,52] These results also explain why the crystallization process of the CE modified E-PBT starts at higher temperatures for all considered cooling rates ϕ. As the secondary crystallization gets more pronounced at higher cooling rates ϕ especially for the CE modified E-PBT (Figure 5b) the growth mechanism changes. Here the Avrami exponent becomes n ≈ 1–2 (one and two dimensional crystal growth) due to growth site impingement, crowding and truncation of spherulites [47,53]. In comparison to the neat E-PBT the spherulite growth gets hindered from its full development. This change in crystal growth also has an impact on the crystallization rate. The corrected crystallization rate constant *k_c_* increases with higher cooling rates ϕ for both neat and CE modified E-PBT, which is in accordance with the tendency of the half-lives τ_1/2_. With higher crystallization rate ϕ of the polymer at higher supercooling the nucleation density increases. However, in the case of the CE modified E-PBT the crystallization rate constant *k_c_* is smaller compared to the neat E-PBT at all cooling rates ϕ. It can be assumed that the crystal growth is hindered by the steric demand (growth site impingement and crowding of spherulites) in such a way that the maximum crystallization is less pronounced and the required time for complete crystallization is longer (Table 2) than for the unmodified PBT beads.

**Figure 6:**
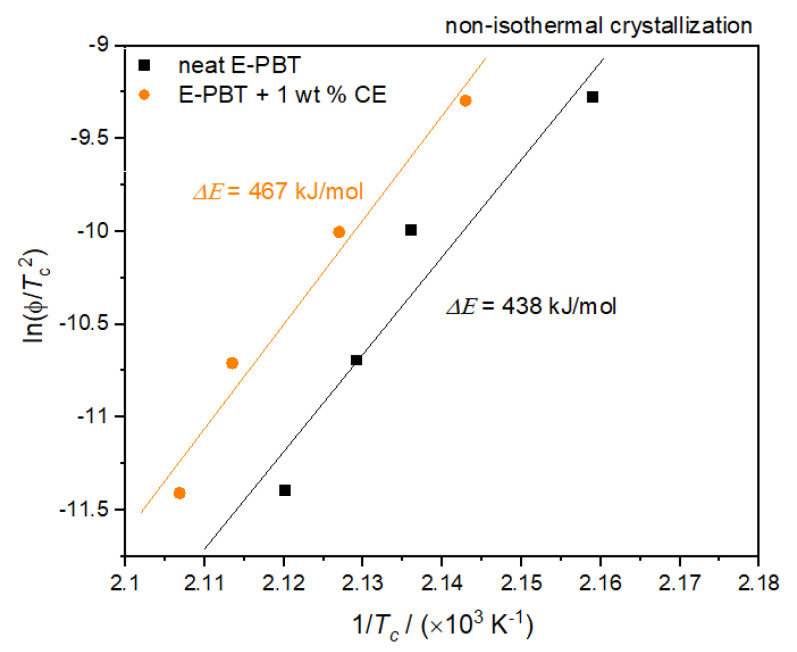
Kissinger Plots of the non-isothermal crystallization of neat and CE modified E-PBT.

The activation energy of the non-isothermal crystallization process of the neat and CE modified E-PBT were calculated by Equations (5) and (6) proposed by Kissinger [54]:

(5) $$\frac{d\left[\ln\left(\phi /T_{c}{}^{2}\right)\right]}{d\left(1/T_{c}\right)}\;=\;-\Delta E/R\;$$

ln(ϕ/*T_c_*^2^) = (1/*T_c_*)⋅(−Δ*E*/*R*) (6)

where Δ*E* is the activation energy of crystallization, *R* is the universal gas constant and *T_c_* is the peak temperature of the crystallization exotherm and ϕ the cooling rate. Plots of ln(ϕ/*T_c_*^2^) versus 1/*T_c_* yield a straight line for neat and CE modified E-PBT which as shown in Figure 6.

According to Equation (6), the values of Δ*E* can be obtained from the slope. Thus, the Δ*E* for neat E-PBT has a value of 438 kJ/mol (slightly higher than the literature [55]) while Δ*E* of 1 wt. % CE modified E-PBT has a value of 467 kJ/mol. The R-square of the fitting line is 0.989 for the neat E-PBT and 0.991 for the CE modified E-PBT. This suggests that the incorporation of branches increases the energy barrier for the non-isothermal crystallization process. This result agrees well with the findings from Avrami approach.

### 3.3. Isothermal Hot-Crystallization Kinetics

During the sintering process, superheated steam is introduced into a cavity in which the bead foams are compressed. To ensure a high quality of interdiffusion between the beads, elevated temperatures and sufficient steaming time are necessary. To investigate the influence of these isothermal conditions on the bead fusion behavior, the isothermal crystallization behavior of the E-PBT beads (neat and CE modified) at the welding temperatures (190 °C–205 °C) (Figure 2) required for E-PBT were determined using DSC. For this purpose, two different routes are examined. The samples were cooled from the melt or heated from the glass to the crystallization temperatures and designated as isothermal hot- or cold-crystallization. The isothermal cold-crystallization route is closer to the steam chest molding process, as the beads are heated from room temperature to the welding temperature and then sintered at a constant temperature. However, in order to obtain a comprehensive understanding of the crystallization processes, both routes are examined and then compared with each other. For both routes the crystalline properties of the outer layers (unmodified and CE modified) are considered since the formation of inter-bead bonding involves the diffusion of polymer chains across the interfaces.

**Figure 7:**
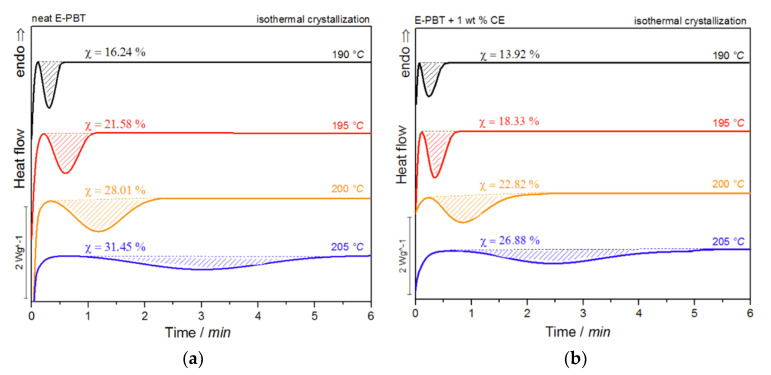
DSC traces of (**a**) neat and (**b**) 1 wt. % CE modified E-PBT isothermally hot-crystallized at different. Crystallization temperatures *T_c_*.

Figure 7a,b show the DSC thermograms of the neat and CE modified PBT beads (outer layers) at various isothermal crystallization temperatures *T_c_*.

**Figure 8:**
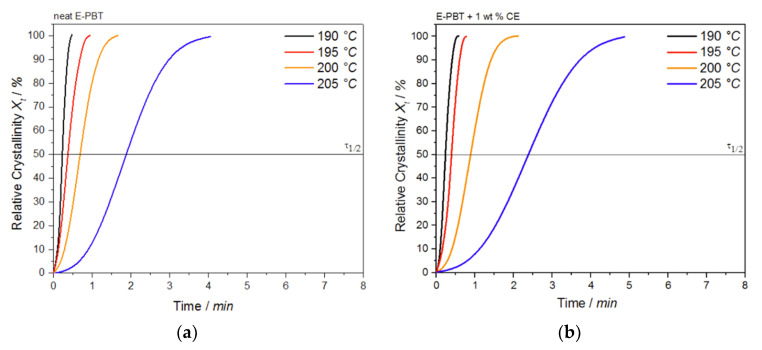
Conversion of the crystallization process at different crystallization temperatures *T_c_* for (**a**) neat and (**b**) 1 wt. % CE modified E-PBT.

For both neat and CE modified PBT beads, the crystallization peak occurs later and gets broader with increasing crystallization temperature *T_c_* (summarized in Table 4). This implies that the crystallization time increases, corresponding to a more pronounced enthalpy of crystallization as the crystallization temperature *T_c_* rises. At the same time, it can be seen that for all crystallization temperatures *T_c_* considered, the crystallization of the CE modified E-PBT is less pronounced, the crystallization process starts earlier and the required time for complete crystallization is slightly longer than for the unmodified PBT beads, which both is in line with the non-isothermal experiments. The earlier start of crystallization for the CE modified E-PBT can be explained by the already mentioned nucleation effect of the branches. With the help of isothermal crystallization exotherms (Figure 7) the relative crystallinity (*X_t_*) at different crystallization times can be obtained using the following equation [56]:

(7) $$X_{t}=\frac{\int_{0}^{t}\;\dot{Q}\left(t^{\prime }\right)\;dt^{\prime }}{\int_{0}^{\infty }\;\dot{Q}\left(t^{\prime }\right)dt^{\prime }}$$

where $\dot{Q}$(*t*^′^) is the heat flow rate. The plot of *X_t_* as a function of time *t* for the neat and CE modified E-PBT undergoing the isothermal hot-crystallization process is shown in Figure 8. The time determination (Figure 8) is done for each curve in such a way that the onset time for crystallization (Figure 7) corresponds to *t* = 0.

Again, all curves show typical sigmoid dependence with time and are linear between 10 to 90%. The required time for complete crystallization for both neat and CE modified E-PBT becomes longer with increasing crystallization temperature *T_c_*, which is in accordance with the tendency of the half-lives τ_1/2_. The half-time of crystallization τ_1/2_ obtained from Figure 8 are listed in Table 4 for neat and CE modified E-PBT.

**Figure 9:**
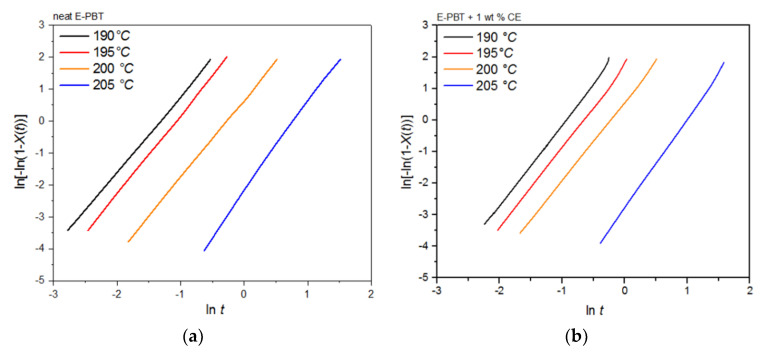
Avrami analysis for (**a**) neat and (**b**) 1 wt. % CE modified E-PBT at different isothermal hot-crystallization temperatures *T_c_*.

It can be clearly observed that the incorporation of branches increases the half-life time τ_1/2_ and decreases the overall crystallization rate of PBT at all crystallization temperatures *T_c_*. In general, there are two opposing factors for the temperature dependence of the growth rate of crystals. The thermodynamic free energy change necessary for the formation of crystal nuclei is the driving force for the crystallization which decreases with increasing crystallization temperature. At the same time, the activation energy for the diffusion of polymer chains increases with rising temperature so that the chains can move toward the surface of the crystal. Further kinetic investigations of the isothermal hot-crystallization were analyzed on the basis of the Avrami approach (Equations (2) and (3)). The Avrami plots of the neat and CE modified E-PBT are shown in Figure 9 and the corresponding Avrami *n* and rate constants *k* yielded by linear regression of these straight lines are listed in Table 5. Neat E-PBT has a linear relationship over the whole crystallization process. In comparison the CE modified E-PBT holds linear relationship up to 90% of relative crystallinity. Above 90% a slight deviation from linearity is observed which can be attributed to secondary crystallization. At a crystallization temperature of 205 °C the Avrami exponent *n* for neat E-PBT is about 2.7, which is in good agreement with previously reported data by other authors [57,58,59] (*n* = 2.6–2.9) indicating a three-dimensional spherulite growth with athermal nucleation mechanism [58,60]. For the crystallization temperatures 190, 195, and 200 °C the Avrami parameter *n* is about 2.4, which is slightly lower than the values reported in the literature. Compared to the neat E-PBT, the CE modified E-PBT has higher Avrami exponents *n* at all observed temperatures varying between *n* = 2.6–2.8 confirming the additional heterogeneous nucleation effect induced by the existing branches. Regarding to the crystallization rate constant *k* two different trends are notable. With increasing crystallization temperature *T_c_* the crystallization rate constant *k* decreases for both neat and CE modified E-PBT which is in agreement with the observed half-lives τ_1/2_ (Table 4). Furthermore, the CE modified samples crystallize more slowly at all given temperatures. As mentioned above for the non-isothermal crystallization considerations, the introduction of branches hinders the chain mobility and thus, retards crystal growth process. Moreover, with higher temperatures the crystal nuclei becomes harder to format which accords with the results given in Figure 7 and Figure 8 and Table 4 and Table 5 and follows the fact that the driving force increases as the polymer is supercooled [50]. The earlier crystallization starts for the CE modified E-PBT at all isothermal crystallization temperatures *T_c_* confirms the additional nucleation effect which was already observed for the non-isothermal crystallization kinetics. However, as the CE modified E-PBT requires more time for complete crystallization (expressing also in the crystallization rate) than the neat E-PBT, it can be assumed that the hindering effect by the existing branches is stronger than their nucleation effect.

**Figure 10:**
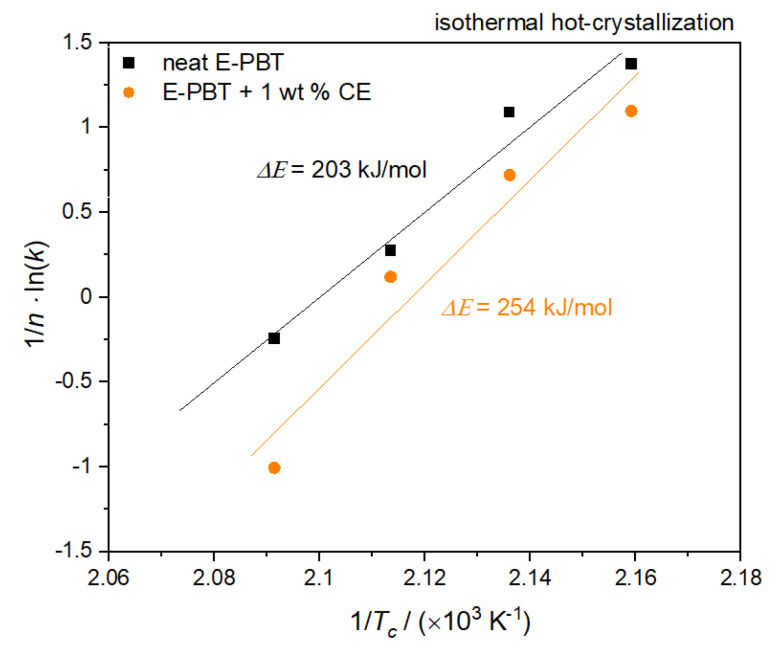
Plot of (1/*n*) ln*k* versus 1/*T_c_* for the determination of the activation energy for the isothermal hot-crystallization of neat and 1 wt. % CE modified E-PBT.

Furthermore, it is possible to determine the activation energy of crystallization since the Avrami parameter *k* is assumed to be thermally activated which can be described by the Arrhenius equation [61] as follows:

*k*^1/n^ = *k*_0_ exp(−Δ*E*/*RT_c_*) (8)

(1/*n*) ln*k* = ln*k*_0_ − (Δ*E*/*RT_c_*) (9)

where *k*_0_ is a pre-exponential factor independent of temperature, Δ*E* is the activation energy and *R* the gas constant. Plots of (1/*n*) ln*k* versus 1/*T_c_* yield a straight line for neat and CE modified E-PBT which are shown in Figure 10.

According to Equation (9), the values of Δ*E* can be obtained from the slope. The calculated activation energy Δ*E* for neat E-PBT is 203 kJ/mol (lower compared to values reported in the literature [50,57]) and 254 kJ/mol for the 1 wt. % CE modified E-PBT. The R-square of the fitting line is 0.987 for the neat E-PBT and 0.986 for the CE modified E-PBT. As already mentioned for the considerations of the activation energy of the non-isothermal crystallization, the incorporation of branches increases the energy barrier. This confirms the results of the Avrami studies elaborated in 3.3, namely that the introduction of branches hinders the chain mobility and thus, retards the crystal growth process.

### 3.4. Isothermal Cold-Crystallization Kinetics

**Figure 11:**
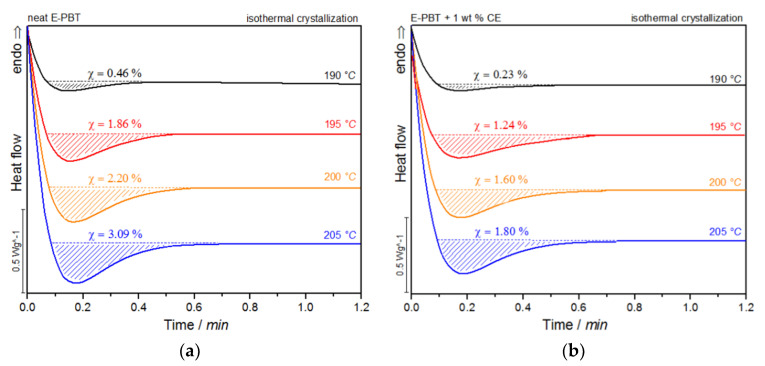
DSC traces of E-PBT (**a**) neat and (**b**) CE modified isothermally cold-crystallized at different crystallization temperatures *T_c_*.

Since the isothermal cold-crystallization route is closer to the actual steam chest mol-ding process, better hypotheses can be derived from this route in order to explain the different phenomena. In accordance with the sintering process the isothermal crystallization behavior of the E-PBT beads (neat and CE modified) were determined at the welding temperatures (190 °C–205 °C) (Figure 11).

For both neat and CE modified PBT beads, the crystallization peak occurs later and becomes broader with increasing crystallization temperature *T_c_*. This implies that the crystallization time increases. Furthermore, a more pronounced enthalpy of crystallization is found as the crystallization temperature *T_c_* rises. At the same time, it can be seen that all crystallization temperatures *T_c_* considered, the crystallization of the CE modified PBT beads is less pronounced and also the required time for complete crystallization is slightly longer than for the neat PBT beads.

**Figure 12:**
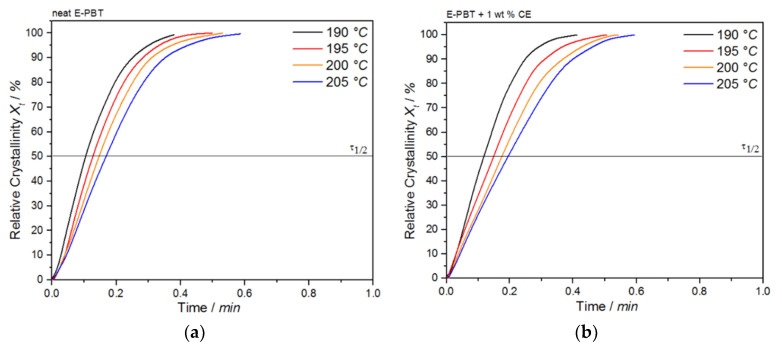
Plot of relative crystallinity *X_t_* as a function of time *t* for (**a**) neat and (**b**) CE modified E-PBT at different crystallization temperatures *T_c_*.

The plot of *X_t_* as a function of time *t* for the neat and CE modified E-PBT undergoing the isothermal hot-crystallization process at various crystallization temperatures *T_c_* is shown in Figure 12.

The required time for complete crystallization for both neat and CE modified E-PBT becomes longer with increasing crystallization temperature *T_c_*, which is accordance with the crystallization half-life times τ_1/2_ (Table 6). As already seen for the isothermal hot-crystallization kinetics the crystallization half-time τ_1/2_ increases with higher temperatures due to the higher chain mobility which retards the crystal growth process. The existing branches in the CE modified E-PBT seem to hinder the overall crystallization process, which can be seen in greater crystallization half times *t*_1/2_ for all considered crystallization temperatures *T_c_*.

**Figure 13:**
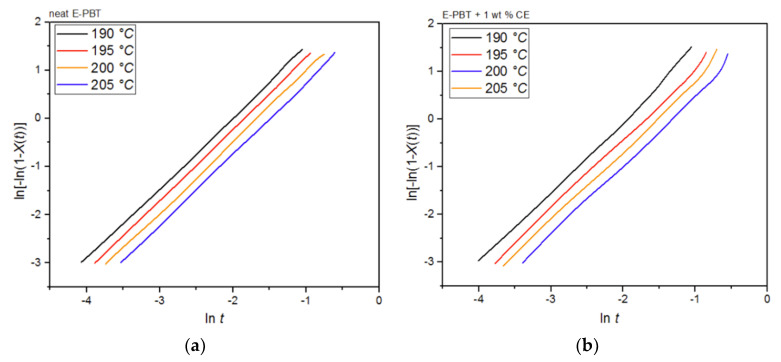
Avrami analysis for (**a**) neat and (**b**) 1 wt. % CE modified E-PBT at various isothermal cold-crystallization temperatures *T_c_*.

Plots of ln[−ln(1 − *X_t_*)] versus ln*t* at given cold-crystallization temperatures *T_c_* yield a straight line for neat and CE modified E-PBT, which are shown in Figure 13a,b. Accor-ding to Equation (3), values of *n* and *k* are obtained from the slope and the point of intersection with the *y*-axis and are listed in Table 7. Both neat and CE modified E-PBT have Avrami parameters in the range of *n* ≈ 1.45 indicating a sporadic nucleation accompanied by a lamellar growth mode. In Section 3.2 we already got insights into the crystallization behavior of PBT during the bead foam process (under-water granulation) via non-isothermal crystallization measurements. Once the PBT bead foams have cooled completely, a certain degree of crystallinity is already present in PBT beads before they are subsequently welded (simulated here by the isothermal cold-crystallization). Since the crystallization of polymers can be divided into two consecutive processes, primary and secondary crystallization [62], it can be assumed that during the isothermal crystallization process shown here, mainly a secondary crystallization process is considered. This would also explain why the Avrami parameters *n* are significantly smaller than shown previously. When the PBT specimens are completely filled with spherulites this secondary crystallization can be understood as an attachment of chain segments to preexisting crystal growth faces, an increase of lamellar thickness, perfection of crystals or the formation of lamellar stacks [62,63,64]. Regarding to the change of the Avrami exponent from 3 (Section 3.2 and Section 3.3) to around 1.45, the crystallization changes to one-dimensional crystal growth due to growth site impingement, crowding and truncation of spherulites [47,53]. This phenomenon could also be analyzed by the Avrami approach done by Xu et al. [62]. The fact that the crystallization rate *k* for the CE modified E-PBT is smaller compared to that one of neat E-PBT at all considered crystallization temperatures can be explained by the already explained hindering effect due to the existing branches (Section 3.2 and Section 3.3). The change of the crystallization rate constant *k* as a function of the temperature is much more pronounced for the isothermal hot-crystallization (Table 5) compared to the cold-crystallization (Table 7). These observations can be attributed to the different crystallization mechanisms (primary and secondary crystallization) caused by the different isothermal crystallization routes. In the case of the isothermal cold-crystallization, crystals are already present at all considered temperatures, from which crystal growth is induced, so that the energy barrier is lowered and the crystallization process is faster.

**Figure 14:**
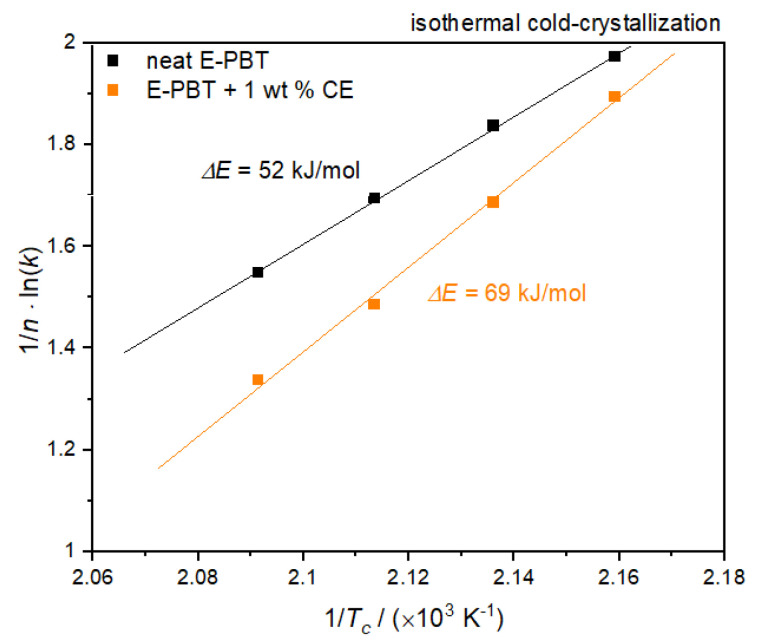
Plot of (1/*n*) ln*k* versus 1/*T_c_* for the determination of the activation energy for the isothermal coldcrystallization of neat and 1 wt. % CE modified E-PBT.

According to Equation (9), the value of the activation energy Δ*E* of neat and CE modified E-PBT can be obtained from the slopes of straight lines in Figure 14.

Also, for the considerations of the activation energy Δ*E* of the isothermal cold crystallization follows that the energy barrier for crystallization process of the CE modified E-PBT is higher (Δ*E* = 69 kJ/mol) compared to the this of the neat E-PBT (Δ*E* = 52 kJ/mol). The R-square of the fitting line is 0.999 for the neat E-PBT and 0.995 for the CE modified E-PBT. Since the isothermal cold-crystallization route is closer to the steam chest molding process, the results obtained are evaluated with regard to the interdiffusion mechanism in E-PBT. The cold-crystallization and the crystallization rate is more pronounced in the unmodified PBT than in the CE modified PBT. These findings are also in line with the initial observations in Figure 2. Following the reasoning that mainly amorphous regions contribute to an interdiffusion of the polymer chains across the bead boundaries and form a physical bond between the beads, it is obvious why the CE modified foamed beads can be welded into a part. In the temperature range where the molding process is realized, unmodified E-PBT crystallizes faster than CE modified one. The longer retention time of the chains causes a slower crystallization behavior, and due to the steric demand of the branches, the maximum crystallization is also less pronounced. In this case, more amorphous regions would remain, which could be contribute to the interbead bonding. So far, it is unclear what kind of chain architecture is initiated by the CE modification. It is of great interest, which chain architecture in PBT is most suitable for realizing the interbead bonding. Thus, this matter will be the subject of further research.

## 4. Conclusions

Classical theories explaining the moldability of EPP fail to describe the behavior of chain extended E-PBT as no double melting peak exists and fusion takes place below the expected temperature range. In our study, detailed differential scanning calorimetry was performed on neat and chain extended E-EBT in order to understand the impact of thermal properties of E-PBT on its molding behavior. No strong difference in crystallinity between the bead outer layer and the center of the bead was found, thus the hypothesis that the outer layer is completely amorphous could be discarded. The investigation of the melting and crystallization behavior of neat and 1 wt. % CE modified E-PBT was done under isothermal and non-isothermal conditions. For both routes it was found that the incorporation of chain extender significantly slows down crystallization and re-crystallization thus allowing the interdiffusion of chain across neighboring beads and thus also crystallization across the bead interface. Therefore, it is concluded that sufficient time for polymer interdiffusion during molding is crucial and requires adjusted crystallization kinetics. So far, it is not clear what kind of chain architecture is initiated by the CE reaction. It is of great interest, which chain architecture in PBT is most suitable for realizing the interbead bonding. Thus, this matter will be the subject of further research.

## Figures and Tables

**Table 1:** Comparison of determined melting temperature T_m_, melting enthalpy ΔH_m_ and relative crystallinity χ of neat E-PBT and 1 wt. % CE modified E-PBT outer layers and cores.

	Neat E-PBT Outer Layer	Neat E-PBT Core	E-PBT + 1 wt. % CE Outer Layer	E-PBT + 1 wt. % CE Core
**T_m_/°C**	224.6	224.1	222.9	222.1
χ **/%**	37.2	32.5	31.8	26.3
Δ ***H_m_*/J/g**	52.1	45.5	44.8	36.9
**T_onset_ − T_offset_/°C**	209–240	212–238	209–233	212–232

**Table 2:** Crystallinity, half-time crystallization, and Avrami range for neat and the CE modified E-PBT.

Neat E-PBT	Crystallinity χ/%	τ_1/2_/min	Avrami Range (*T_onset_* − *T_offset_*)
−2.5 K/min	39.17	2.00	206–190 °C
−5 K/min	38.85	1.30	203–185 °C
−10 K/min	36.25	0.70	201–171 °C
−20 K/min	34.21	0.45	199–158 °C
**E-PBT + 1 wt. % CE**			
−2.5 K/min	36.71	2.30	208–187 °C
−5 K/min	36.31	1.60	205–182 °C
−10 K/min	35.65	0.85	203–163 °C
−20 K/min	32.70	0.55	201–155 °C

**Table 3:** Avrami exponent n, nucleation, and growth rate constant k_c_ at different cooling rates ϕ for neat and the CE modified E-PBT.

Neat E-PBT	*n*	*k_c_* (min^−^*^n^*)
−2.5 K/min	3.23	0.34
−5 K/min	3.46	0.76
−10 K/min	3.44	1.08
−20 K/min	3.42	1.16
**E-PBT + 1 wt. % CE**		
−2.5 K/min	4.28	0.19
−5 K/min	4.24	0.63
−10 K/min	4.10	0.99
−20 K/min	3.95	1.08

**Table 4:** Crystallinity and half-time crystallization at different isothermal hot-crystallization temperatures for neat and the CE modified E-PBT.

Neat E-PBT	Crystallinity χ/%	τ_1/2_/min
190 °C	16.24	0.23
195 °C	21.58	0.37
200 °C	28.01	0.65
205 °C	31.45	1.80
**E-PBT + 1 wt. % CE**		
190 °C	13.92	0.25
195 °C	18.33	0.37
200 °C	22.82	0.90
205 °C	26.88	2.40

**Table 5:** Avrami exponent n, nucleation and growth rate constant k at different isothermal hot-crystallization temperatures *T_c_* for neat and the CE modified E-PBT.

Neat E-PBT	*n*	*k* (min*^−n^*)
190 °C	2.38	24.87
195 °C	2.30	14.04
200 °C	2.40	1.93
205 °C	2.70	0.52
**E-PBT + 1 wt. % CE**		
190 °C	2.60	13.80
195 °C	2.60	6.52
200 °C	2.70	1.69
205 °C	2.80	0.06

**Table 6:** Comparison of determined crystallization χ and half lives t_1/2_ at different isothermal crystallization temperatures *T_c_*.

Neat E-PBT	Crystallinity χ/%	τ_1/2_/min
190 °C	0.46	0.10
195 °C	1.86	0.13
200 °C	2.20	0.15
205 °C	3.09	0.17
**E-PBT + 1 wt. % CE**		
190 °C	0.23	0.12
195 °C	1.24	0.15
200 °C	1.60	0.18
205 °C	1.80	0.20

**Table 7:** Avrami parameters of the isothermal crystallization for neat and 1 wt. % CE modified E-PBT.

Neat E-PBT	*n*	*k*/min*^-n^*
190 °C	1.45	17.46
195 °C	1.47	14.88
200 °C	1.47	12.06
205 °C	1.46	9.58
**E-PBT + 1 wt. % CE**		
190 °C	1.42	14.73
195 °C	1.37	10.07
200 °C	1.42	8.25
205 °C	1.42	6.68

## Data Availability

The data presented in this study are available on request from the corresponding author.
